# A^2^G-SRNet: An Adaptive Attention-Guided Transformer and Super-Resolution Network for Enhanced Aircraft Detection in Satellite Imagery

**DOI:** 10.3390/s25216506

**Published:** 2025-10-22

**Authors:** Nan Chen, Biao Zhang, Hongjie He, Kyle Gao, Zhouzhou Liu, Liangzhi Li

**Affiliations:** 1 College of Computer Science, Xihang University, Xi’an 710077, China; chcdut@126.com (N.C.); liuzhouzhou8192@126.com (Z.L.); 2 Key Laboratory of Smart Earth, No. 8 Minzu Yuan Road, Beijing 100029, China; 3 Aerial Photogrammetry and Remote Sensing Group Co., Ltd., Xi’an 710100, China; 4 Department of Geography and Environmental Management, University of Waterloo, Waterloo, ON N2L 3G1, Canada; hongjie.he@uwaterloo.ca; 5 Department of Systems Design Engineering, University of Waterloo, Waterloo, ON N2L 3G1, Canada; y56gao@uwaterloo.ca; 6 Xi’an Key Laboratory of Territorial Spatial Information, Chang’an University, Xi’an 710064, China

**Keywords:** aircraft detection, super-resolution, global-local adaptive detection, saliency-aware attention, small object detection

## Abstract

Accurate aircraft detection in remote sensing imagery is critical for aerospace surveillance, military reconnaissance, and aviation security but remains fundamentally challenged by extreme scale variations, arbitrary orientations, and dense spatial clustering in high-resolution scenes. This paper presents an adaptive attention-guided super-resolution network that integrates multi-scale feature learning with saliency-aware processing to address these challenges. Our architecture introduces three key innovations: (1) A hierarchical coarse-to-fine detection pipeline that first identifies potential regions in downsampled imagery before applying precision refinement, (2) A saliency-aware tile selection module employing learnable attention tokens to dynamically localize aircraft-dense regions without manual thresholds, and (3) A local tile refinement network combining transformer-based super-resolution for target regions with efficient upsampling for background areas. Extensive experiments on DIOR and FAIR1M benchmarks demonstrate state-of-the-art performance, achieving 93.1% AP_50_ (DIOR) and 83.2% AP_50_ (FAIR1M), significantly outperforming existing super-resolution-enhanced detectors. The proposed framework offers an adaptive sensing solution for satellite-based aircraft detection, effectively mitigating scale variations and background clutter in real-world operational environments.

## 1. Introduction

Remote sensing image object detection has emerged as a critical research focus in optical remote sensing image processing, with aircraft detection playing a pivotal role in aerospace monitoring, military reconnaissance, aviation safety, and unmanned aerial vehicle (UAV) surveillance [1]. In military operations, aerial and remote sensing images are indispensable for monitoring aircraft movements in military bases and airports, providing critical intelligence for strategic planning and battlefield decision-making [2]. Rapid and precise identification of enemy aircraft deployment, takeoff and landing patterns, and spatial distribution can significantly enhance situational awareness and operational effectiveness. Advances in remote sensing satellite technology and optical imaging systems have enabled the acquisition of high-resolution aerial imagery, facilitating large-scale, uninterrupted, and high-precision aircraft detection [3,4]. Despite significant progress in deep learning-based object detection algorithms, challenges persist in detecting remote sensing aircraft due to their small size, arbitrary orientations, inter-class similarity among different aircraft models, and fine-grained intra-class variations [5]. Consequently, achieving accurate, efficient, and real-time detection along with fine-grained recognition of aircraft remains a challenging yet crucial research direction.

**Figure 1:**
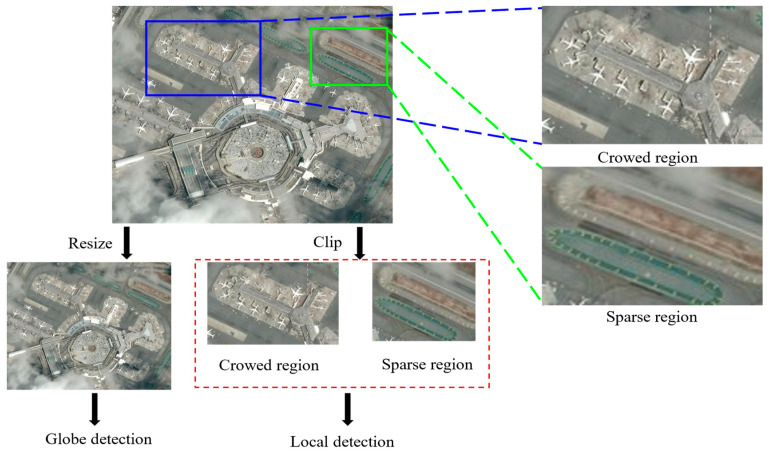
Aircraft detection challenges in remote sensing: Small-scale degradation and non-uniform distribution.

As shown in Figure 1, high-resolution remote sensing data introduces significant challenges for aircraft detection, including densely clustered targets, extreme scale variations, and complex background clutter. These complexities demand advanced analytical techniques to ensure accurate detection and classification. The primary challenges can be summarized as follows: (1) Scale variation—most aircraft appear at extremely small scales relative to the full image, and when high-resolution images are downsampled to standard network input sizes, critical features degrade severely, making recognition difficult; (2) Spatial distribution imbalance—aircraft are unevenly distributed across airport areas, with dense clusters in parking zones and sparse occurrences elsewhere. Traditional uniform cropping methods inefficiently process empty regions while often truncating aircraft at crop boundaries, further degrading detection performance.

Building upon the success of convolutional neural networks (CNNs) in natural image detection, researchers have made significant efforts to adapt these methods for aircraft detection in remote sensing imagery. The strong inductive biases of CNNs, particularly their translation equivariance and locality, made them exceptionally well-suited for extracting hierarchical features from the textured and structured patterns prevalent in overhead imagery. Recent research efforts have yielded significant methodological innovations to address the persistent challenges in remote sensing aircraft detection. For instance, Hu et al. [6] combined saliency detection with deep CNNs, using background priorities to generate fewer but more accurate proposal regions before fine-tuning detection results. Shi et al. [7] developed DPANet, which employs deconvolution and position attention mechanisms to better capture both the external contours and internal structures of aircraft. For weakly supervised learning scenarios where manual annotation proves prohibitively expensive, AlexNet-WSL [8] demonstrates how image-level annotations can be effectively leveraged for aircraft detection. The problem of small target detection in cluttered environments has been addressed through multiscale fusion architectures like MFPN [9], which enhances feature representation by integrating information from multiple receptive fields. For high-resolution imagery containing dense aircraft clusters, SCMask R-CNN [10] extends the popular Mask R-CNN framework with specialized components to improve detection accuracy in complex scenes. The adaptation of existing detection frameworks has shown promise, with YOLO-based approaches [11] offering efficient solutions for satellite imagery characterized by diverse object variations and complex backgrounds. Meanwhile, novel paradigms like X-LineNet [12] reformulate the detection task through a bottom-up approach, modeling aircraft as collections of intersecting line segments to better capture structural characteristics. The deformable convolution paradigm introduced by Ren et al. [13] significantly advanced geometric modeling, with their Deformable ResNet-based Faster R-CNN achieving superior performance in handling aircraft pose variations. Liu et al. [14] proposed a hybrid aircraft detection method that combines corner-based mean-shift clustering for efficient region proposal with CNN-based classification. These methodological advancements collectively represent significant progress in addressing the fundamental challenges of scale variation, orientation diversity, and background complexity. Additionally, rotation-invariant CNN architectures [15] have been proposed to handle the arbitrary orientations of aircraft in overhead imagery, demonstrating improved robustness to viewpoint variations. However, the detection of aircraft in remote sensing imagery presents distinct challenges that conventional CNN architectures struggle to fully address. Unlike natural images, remote sensing data is characterized by top-down perspectives, complex backgrounds, and significant spectral and geometric similarities between targets and surrounding objects. The inherent locality of convolutional operations, while a strength for texture analysis, can limit the model’s ability to capture long-range contextual relationships that are crucial for disambiguating targets from clutter in large-area scenes. Additionally, atmospheric interference and sensor noise further degrade image quality, obscuring critical details necessary for accurate detection.

Recently, the advent of Vision Transformers has introduced a new paradigm for computer vision, offering a powerful mechanism for modeling global dependencies through self-attention. This capability is particularly advantageous for remote sensing image understanding, where the semantic interpretation of a target often depends on its surrounding context (e.g., an aircraft’s relationship to a runway or hangar). This has led to the development of hybrid CNN-Transformer architectures and pure Transformer-based models tailored for remote sensing tasks. For example, frameworks like FCIHMRT [16] have demonstrated the effectiveness of Transformer encoders for multi-level feature interaction and refinement in high-resolution satellite imagery. Similarly, the adaptation of detectors like DETR [17] and the use of hierarchical vision backbones like Swin Transformer have shown promise in capturing global context more effectively than traditional CNNs. DETR [17] marked a paradigm shift by pioneering transformers and self-attention mechanisms into object detection, reformulating it as a set prediction problem and eliminating traditional post-processing steps. While innovative, DETR suffered from slow convergence and limited small object detection capability. Subsequent improvements addressed these limitations: Deformable DETR [18] enhanced convergence through localized attention mechanisms, UP-DETR [19] improved accuracy via unsupervised pre-training, and Efficient DETR [20] strengthened decoder queries by selecting Top-K positions from encoder predictions. However, these enhancements often increased computational complexity due to stacked encoder–decoder architecture. Parallel to these architectural advancements, specialized attention mechanisms have been developed to address the challenges of aircraft detection in remote sensing imagery. Zhao et al. [21] proposed a dilated attention block that effectively enhances the learning of aircraft scattering features while filtering out irrelevant background information. Luo et al. [22] introduced an Efficient Bidirectional Path Aggregated Attention Network (EBPA2N) that employs an involution operator combined with a shuffle attention mechanism. This design enables refined suppression of redundant features while preserving critical aircraft characteristics, resulting in both high detection rates and low false alarm rates (FAR) in complex airport environments. Complementing these attention-based approaches, Zhu et al. [23] tackled the challenges of representing weak and small objects and resolving overlapping detection boxes through multilayer feature fusion and an improved nonmaximal suppression algorithm. Their method employs transfer learning with region-based CNNs, integrates L2 norm normalization with feature connection and scaling for effective feature fusion, and introduces a soft decision function in nonmaximal suppression to handle detection box overlaps, thereby enhancing airplane detection performance in airport areas.

Our work builds upon this progression by integrating a Transformer-based saliency and refinement mechanism within a CNN detection framework, aiming to synergistically combine the local feature precision of CNNs with the global contextual reasoning of Transformers, while maintaining computational efficiency through selective processing of critical regions.

Despite these innovations, critical limitations remain that hinder operational deployment. Current methods still struggle with consistent detection of extremely small aircraft targets, particularly when their visual signatures are degraded by sensor noise or atmospheric interference. The problem of false alarms induced by background clutter persists, especially in heterogeneous airport environments where numerous man-made objects share visual similarities with aircraft. Furthermore, the computational efficiency of many advanced methods remains insufficient for real-time processing of high-resolution remote sensing data streams. These unresolved challenges highlight the need for continued innovation in remote sensing aircraft detection. The ideal solution must simultaneously address three key requirements: (1) robust detection of sub-20px targets in noisy environments, (2) effective suppression of false alarms in complex scenes, and (3) computationally efficient processing to enable real-time operation.

To address these challenges, the paper presents an adaptive attention-guided super-resolution network (A^2^G-SRNet) that integrates multi-scale feature learning with a saliency-aware processing framework that enhances detection accuracy by integrating super-resolution reconstruction with multi-scale feature learning. Our approach leverages the self-attention mechanism of Transformers to capture long-range dependencies and contextual information while integrating a global-local adaptive super-resolution module to enhance small and densely packed aircraft regions. We first conduct global coarse detection on the down-sampled image to roughly recognize the target aircraft objects, which extracts the outline clues of the entire airport scene. To accurately locate the crowded regions where aircraft are densely packed, we propose a Saliency-Aware Tile Selection (SATS) module that automatically identifies critical regions through a cross-attention mechanism with learnable tokens. This innovative design eliminates manual threshold tuning and dynamically adapts to varying aircraft densities upsampling. The refined regions are analyzed by the local detector, with final detections fused via NMS. Before feeding into the local fine detector, the selected high-potential subregions first go through a Local Tile Refinement (LTR) network to obtain more detailed information at the pixel level. Then, the enhanced subregions are fed into the local fine detector to gain more accurate results. The final detections are acquired by merging the two detection results with non-maximum suppression (NMS).

Consequently, the salient contributions of this work could be summarized as follows:

- A hierarchical global-local detection framework that employs a coarse-to-fine strategy for multi-scale aircraft detection in complex airport scenes. The framework first performs coarse detection on downsampled images to identify potential regions, then adaptively refines detection for small and densely clustered aircraft targets.
- A Saliency-Aware Tile Selection (SATS) module that leverages learnable attention mechanisms to dynamically identify critical aircraft regions. This innovation eliminates the need for manual threshold tuning while ensuring compatibility with standard detection architectures.
- A Transformer-based Local Tile Refinement (LTR) network that performs selective super-resolution (SR) exclusively on identified aircraft-dense regions through multi-scale feature fusion and attention-guided upsampling, while bypassing computational processing for non-target areas to maintain efficiency.

The rest of this paper is organized as follows. Section 2 reviews related work on object detection and SR methods. Section 3 presents our proposed A^2^G-SRNet framework. Section 4 describes the experiments and results. Finally, Section 5 concludes the paper.

## 2. Related Work

### 2.1. Object Detection Based on Deep Learning

Recent advances in CNN technology have significantly propelled the development of object detection techniques. Object detection algorithms based on deep learning are broadly classified into two categories: one-stage methods and two-stage methods. One-stage detectors bypass explicit region proposals, performing direct classification and regression on densely sampled anchors, offering faster inference at the potential cost of accuracy. Key models include Single Shot MultiBox Detector (SSD) [24], which assigns scores to default boxes on multi-scale feature maps but relies on manual parameter tuning. You Only Look Once (YOLO) series [25] treats detection as a regression problem, simplifying network architecture for real-time performance. YOLOv5 incorporates Generalized Intersection over Union (GIoU) loss and Adam optimization for better handling of densely occluded objects, while YOLOv8 introduces convolutional layers and anchor-free heads for further speed gains. RetinaNet [26] addresses class imbalance with focal loss, improving performance on hard examples, though it struggles with real-time detection of small or multiple objects. Two-stage detectors typically involve a region proposal stage followed by classification and bounding box regression. Pioneering works include Region-based Convolutional Neural Networks (R-CNN) [27], which employs selective search for region proposals and CNNs for feature extraction, with final classification via Support Vector Machines (SVMs). Fast R-CNN [28] improves efficiency by introducing a Region of Interest (RoI) pooling layer to handle scale variations, enabling end-to-end training. Faster R-CNN [29] further enhances speed by integrating a Region Proposal Network (RPN) to generate candidate boxes directly from feature maps, replacing computationally expensive selective search algorithms.

These models have been widely adapted for remote sensing applications. For instance, Ren et al. [30] modified Faster R-CNN by altering the RPN and incorporating contextual information to better detect small objects in remote sensing images. Tang et al. [31] introduced a hyper region proposal network and a cascade boosted classifier to improve recall and reduce false positives through hard negative mining. Yang et al. [32] further advanced small object detection by proposing rotation dense feature pyramid networks (R-DFPN) within a modified Faster R-CNN framework, enhancing the detection performance for both large and small objects. For multimodal imagery, Manish et al. [33] developed a real-time detection framework leveraging mid-level fusion to merge data from multiple modalities, improving robustness across sensor types. In forestry and agriculture, Faster R-CNN has been applied to detect conifer seedlings from drone imagery across seasons and palm trees from satellite data using sliding window techniques [34]. Remote sensing-specific two-stage frameworks address rotation and orientation issues. The RoI Transformer [35] rotates horizontal anchor boxes using fully connected layers for initial proposals, followed by feature extraction for refined regression. SCRDet [36] employs attention mechanisms to mitigate background noise and enhance detection of crowded or small objects. While these advances have significantly improved object detection in remote sensing imagery, persistent challenges in small aircraft detection—particularly under extreme scale variations, dense clustering, and background clutter—motivate the development of specialized frameworks that integrate super-resolution with the detection task.

### 2.2. Object Detection Based on SR

Recent advances in SR techniques have demonstrated significant potential for enhancing small object detection performance in remote sensing applications. Traditional approaches typically employ SR as a preprocessing step, where generative adversarial networks (GANs) are commonly utilized to reconstruct high-resolution images from low-resolution inputs before detection. For instance, Hong et al. [37] introduced a cycle-consistent GAN structure as an SR network coupled with a modified Faster R-CNN architecture, specifically enhancing vehicle detection performance in aerial imagery. Courtrai et al. [38] developed a GAN-based SR method that effectively improved detection accuracy by generating enhanced HR images for subsequent processing. Similarly, Rabbi et al. [39] incorporated a Laplacian operator to extract edge features during SR reconstruction, which notably boosted object localization and classification performance. These methods have proven particularly valuable for detecting aircraft and other small objects in very-high-resolution (VHR) satellite imagery, achieving notable improvements even at challenging resolution levels of 15–30 cm/pixel.

However, such preprocessing-based SR approaches inevitably introduce substantial computational overhead due to the need for full-image super-resolution. To address this limitation, recent works have explored more efficient integration strategies. Wang et al. [40] proposed an innovative SR module that maintains HR representations while processing LR inputs, significantly reducing computational costs in segmentation tasks. Alternative approaches have focused on end-to-end joint optimization frameworks, such as the triple-network architecture combining SR-GAN, edge enhancement, and detector networks proposed in [37], which demonstrated improved performance through gradient backpropagation of detection losses. Despite these advancements, current SR-assisted detection methods still face several critical challenges. First, the reconstruction quality for extremely small objects (e.g., sub-20px targets) remains inconsistent, particularly under conditions of sensor noise or atmospheric interference [41]. Second, the computational demands of many SR-based approaches remain prohibitive for real-time processing of high-resolution remote sensing data streams [42]. Third, most existing methods process entire images at high resolution, failing to focus computational resources on regions of interest. These limitations highlight the need for more sophisticated SR integration strategies that can selectively enhance relevant image regions while maintaining computational efficiency.

## 3. Methodology

**Figure 2:**
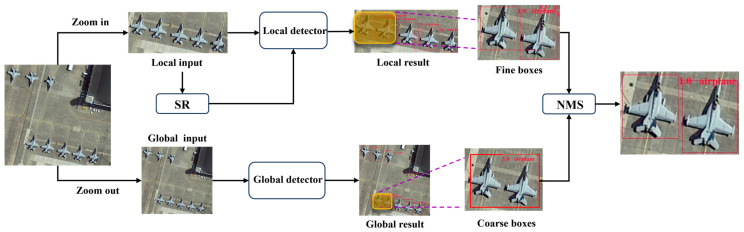
The pipeline of our A^2^G-SRNet network for aircraft detection.

This section presents the proposed A^2^G-SRNet framework for efficient aircraft detection in remote sensing imagery, as shown in Figure 2. Firstly, we introduce the global-local coarse-to-fine detection network, which hierarchically processes high-resolution images through global scene analysis and local region refinement. Subsequently, we detail the saliency-aware tile selection module, an adaptive attention mechanism for identifying critical aircraft-dense regions without manual intervention. Finally, we describe the transformer-based local tile refinement network, which integrates super-resolution reconstruction with multi-scale feature learning to enhance small aircraft details.

### 3.1. The Global-Local Coarse-to-Fine Detection Network

The proposed A^2^G-SRNet framework introduces an end-to-end solution for robust aircraft detection in high-resolution remote sensing imagery. At its core, the system implements a sophisticated coarse-to-fine detection strategy that intelligently allocates computational resources while maintaining high detection accuracy across varying target scales. The framework begins with global coarse detection performed on downsampled full images, where a lightweight backbone network efficiently processes the entire scene to generate preliminary aircraft proposals and establish the overall airport layout. This initial phase serves dual purposes: it provides crucial contextual information about the scene while rapidly identifying potential regions of interest for further analysis. Building upon these coarse detections, our innovative SATS module employs learnable attention mechanisms to automatically identify and extract critical sub-regions containing small or densely clustered aircraft targets. Unlike conventional approaches that rely on manual thresholding or fixed sliding windows, the SATS module dynamically adapts to varying target distributions through a cross-attention mechanism that evaluates both spatial and feature-domain relationships. This intelligent region selection process ensures computational efficiency by focusing subsequent processing only on the most relevant image areas. The selected high-potential regions then undergo targeted enhancement through our LTR network, a Transformer-based architecture specifically designed for adaptive super-resolution. The LTR network combines multi-scale feature extraction with attention-guided upsampling to recover fine-grained details while suppressing noise and background clutter. By operating selectively on critical regions rather than the entire image, this approach achieves significant computational savings while substantially improving the detectability of small targets. The enhanced sub-regions are processed by a local detection head that shares parameters with the global detector but operates on the refined high-resolution features to achieve precise localization. Finally, the framework employs an adaptive fusion mechanism that intelligently combines detection results from both global and local processing stages. Through optimized Non-Maximum Suppression (NMS), the system effectively reconciles the broad contextual understanding from the global phase with the fine-grained localization accuracy from the local phase.

We formalize the aircraft detection bounding boxes as $B^{f}=\left\{\left(b_{k}^{f},p_{k}^{f}\right)\right\}$, where $b_{k}^{f}=\left(x_{1},y_{1},x_{2},y_{2}\right)$ denotes the coordinates of the k−th aircraft bounding box, $p_{k}^{f}\in \left[0,1\right]$ represents the detection confidence score, and the superscript f indicates the final result. Given an input aerial image I, our A^2^G-SRNet network operates through:

(1) $$B^{f}=NMS\left(Detect(I),Detect\left(TR(TS(I))\right)\right)$$

Where $TS\left(\cdot \right)$ denotes our tile selection module that identifies critical aircraft regions, $TR\left(\cdot \right)$ represents the tile refinement process for super-resolution. Critical regions containing densely clustered or small aircraft are then identified by the SATS module, which leverages learnable attention to dynamically crop high-potential sub-images without manual intervention. These selected regions undergo high-fidelity super-resolution via our LTR network, and the refined sub-regions are analyzed by the local detector to generate high-precision predictions. Final detections are obtained by adaptive fusion of global and local results through NMS, ensuring robustness to scale variations while maintaining detection coherence across resolution boundaries.

### 3.2. Saliency-Aware Tile Selection Algorithm

#### 3.2.1. Theoretical Analysis of Saliency-Aware Tile Selection

Drawing from Gestalt theory (e.g., principles of proximity and continuity), SATS groups aircraft-dense tiles based on spatial and feature similarities, treating the image as an organized whole rather than uniform grids. This reduces fragmentation issues in traditional methods, where overly large blocks dilute small object features and small blocks increase computation on empty areas.

SATS is inspired by human visual attention mechanisms, which prioritize salient regions in complex scenes to reduce cognitive load. In remote sensing imagery, where aircraft targets exhibit non-uniform distributions, SATS mimics bottom-up saliency detection by dynamically allocating attention to high-density regions. Theoretically, this aligns with saliency map models, where a topographical representation encodes stimulus conspicuity by integrating multi-scale features (orientation, intensity, etc.). In our case, the multi-scale pyramid structure ensures hierarchical processing, preserving over 95% of candidate tiles with valid objects while minimizing false negatives.

The core of SATS lies in injecting saliency guidance into the Swin Transformer’s windowed self-attention

(2) $$A^{\prime }=A+\beta \cdot S_{w},\;\;\mathrm{where}\;S_{w}=\mathrm{align}(S,w)$$

where A is the original attention score matrix, S is the saliency map (normalized via SoftMax on a two-channel output from a lightweight convolutional layer), $S_{w}$ is the spatially aligned saliency within attention window w (size M), and β is a learnable bias parameter. This additive bias theoretically enhances attention weights for salient (foreground) positions while suppressing background. By incorporating saliency as a local bias, we derive that the modified attention prioritizes queries/keys in salient regions, increasing the probability mass on aircraft-dense tokens. This can be analyzed via information theory: the saliency bias reduces entropy in attention distributions over non-salient areas, focusing computational resources on regions with higher information density. Quantitatively, this lowers the risk of feature degradation in down sampled inputs, as salient tiles are propagated with preserved details.

#### 3.2.2. Architecture and Workflow of Saliency-Aware Tile Selection

**Figure 3:**
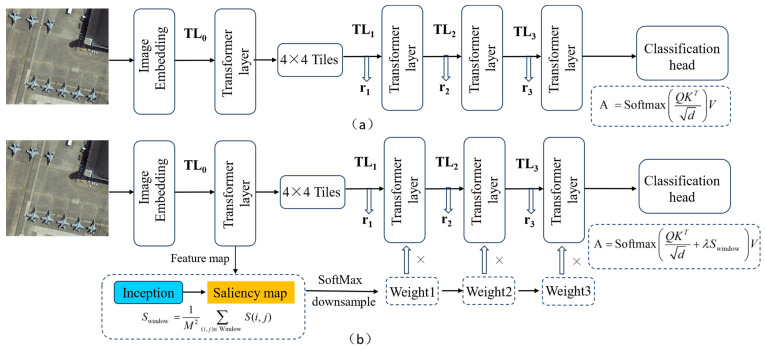
The network architecture of the saliency-aware tile selection algorithm. (**a**) Traditional algorithms divide the input image into uniform grids of fixed size; (**b**) Our proposed algorithm employs a pixel attention network for saliency-based weighting.

As shown in Figure 3, the tile selection module comprises an image encoder followed by multiple tile classifiers. First, the embedding layer and initial transformer layer of the encoder transform the LR input image $I_{LR}\in {\mathbb{R}}^{H\times W\times C}$ (H, W and C are the input LR image height, width and the number of channels) into feature representations $r_{0}\in {\mathbb{R}}^{(H/p)\times (W/p)\times C}$. where p is the patch size of each token and C is the number of channels for each token. After that, three transformer layers $TL_{1}$, $TL_{2}$, and $TL_{3}$ generate representations of these tokens at three different scales. The transformer layer adopts the structure of Swin Transformer. In addition, with the feature merging layer, we can obtain features at various scales to enable the implementation of pyramid structure as follows, $r_{1}=TL_{1}\left(r_{0}\right)$, $r_{2}=TL_{2}\left(r_{1}\right)$, $r_{3}=TL_{3}\left(r_{2}\right)$, where each token in the multi-scale representations corresponds to tiles of size 2p×2p ($r_{1}$), 4p×4p ($r_{2}$) and 8p×8p ($r_{3}$), respectively, preserving the original 2× scaling ratios while enabling content-aware initial divisions. Traditional block-based methods typically divide input images into uniform grids, processing each grid unit independently through fixed-size partitions. While computationally efficient, this uniform treatment fails to account for the non-uniform distribution of aircraft targets in remote sensing imagery, often resulting in either excessive computation on empty regions or insufficient resolution for clustered small aircraft-overly large blocks may cause loss of small object features, while excessively small blocks become fragmented. To more effectively capture aircraft targets against complex background, our saliency-aware hierarchical tile selection module addresses these limitations through an adaptive attention mechanism that intelligently allocates computational resources based on target density and spatial importance. Our proposed algorithm employs a pixel attention network for saliency-based weighting: the process starts with the input low-resolution image undergoing patch embedding and Transformer encoding, followed by multi-scale feature merging; saliency maps are generated via a multi-scale Inception module and lightweight convolution, then injected as biases into windowed self-attention for dynamic tile prioritization.

Specifically, the input feature map $r_{0}$ is first processed through a multi-scale inception module, which employs parallel convolutional kernels with varying receptive fields to capture hierarchical contextual features. The multi-scale Inception module is adapted from the original Inception architecture [43] to efficiently extract features at multiple scales while controlling computational cost through dimension reduction. It consists of four parallel branches: (1) a 1 × 1 convolution (64 filters) for point-wise feature integration and channel reduction; (2) a 1 × 1 convolution (48 filters) followed by a 3 × 3 convolution (64 filters) for local context capture; (3) a 1 × 1 convolution (32 filters) followed by a 5 × 5 convolution (48 filters) for broader receptive fields; and (4) a max-pooling layer (3 × 3 kernel, stride 1) followed by a 1 × 1 convolution (32 filters) to incorporate non-linear pooling information. All convolutions use ReLU activation and same-padding to preserve spatial dimensions. The outputs from these branches are concatenated along the channel dimension, resulting in a fused feature map with 208 channels. This design reduces parameters compared to a single large-kernel convolution by approximately 33%, as the 1 × 1 convolutions act as bottlenecks for dimensionality reduction before larger kernels. The varying kernel sizes (1 × 1, 3 × 3, 5 × 5) ensure multi-scale feature extraction, aligning with the non-uniform aircraft distributions in RSIs, where small kernels capture fine details (e.g., aircraft edges) and larger ones model contextual clutter.

**Figure 4:**
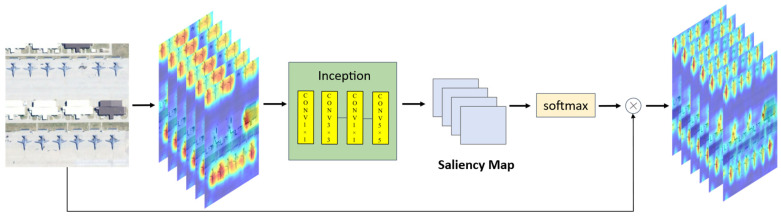
Process of enhancing feature map attention using saliency maps.

Subsequently, a lightweight convolutional layer generates a two-channel saliency map $S\in {\mathbb{R}}^{2\times H\times W}$, where each channel corresponds to the foreground (target) and background activation scores, respectively. To normalize the saliency weights, a SoftMax function is applied along the channel dimension, ensuring the spatial attention values lie within the range [0, 1], as shown in Figure 4. Subsequently, we downsample the saliency maps to match feature dimensions at different Transformer layers ($r_{1}$, $r_{2}$ and $r_{3}$) using bilinear interpolation, and then we inject saliency guidance in Swin Transformer’s windowed self-attention as follows:

(3) $$\;\mathrm{Attention}\;=\mathrm{Softmax}\left(\frac{QK^{T}}{\sqrt{d}}+\lambda S_{\mathrm{window}\;}\right)V,\;S_{\mathrm{window}\;}=\frac{1}{M^{2}}\sum_{(i,j)\in \;\mathrm{Window}\;}S(i,j)$$

where S(i,j) is saliency score at spatial position (i,j). $S_{\mathrm{window}\;}$ is spatially aligned to corresponding attention windows. $\lambda S_{\mathrm{window}\;}$ acts as a local bias to attention scores, enhancing target regions while preserving background information. M×M is window size.

Subsequently, each of the three hierarchical features undergoes independent processing through a multi-layer perception (MLP) and a Gumbel-SoftMax layer. The pyramid supervision signal is constructed through adaptive max-pooling of instance segmentation masks at three different scales (1/4, 1/8, and 1/16 of the original resolution), generating corresponding binary labels $y_{i}$ to guide the training of the tile selection module. Positive labels are assigned when tiles satisfy either criterion: (1) geometric containment of at least one object centroids, or (2) IoU > 0.5 with ground truth annotations. This hierarchical labeling framework achieves two key objectives: (1) it preserves over 95% of candidate tiles containing valid object instances, while (2) systematically minimizing false negative detections through multi-scale verification. The processed tiles are then ultimately stratified into two distinct categories based on their classification confidence scores: (1) high-probability positive tiles (confidence > 0.8) containing valid object instances, and (2) definitive negative background tiles (confidence < 0.2). This binary partitioning scheme ensures that only reliably classified proposals are propagated to the refinement module for subsequent high-precision processing.

### 3.3. Local Tile Refinement Network

**Figure 5:**
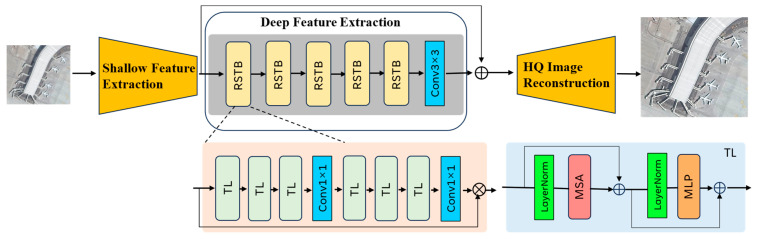
The structure of the lightweight transformer-based superresolution.

Based on the classification in Section 3.2, there are two types of tiles: Positive Tile Refinement (PTR) targeting object-containing tiles, and Negative Tile Refinement (NTR) for tiles with solely background pixels. For positive tiles which contain aircraft targets, we adopt a lightweight transformer-based process for deep feature extraction and image reconstruction. Specifically, for a given tile $T_{LR}\in {\mathbb{R}}^{8p\times 8p\times C}$ the convolution layer first extracts the shallow feature $F_{S}\in {\mathbb{R}}^{8p\times 8p\times C_{f}}$, where $C_{f}$ is the number of channels for features. Subsequently, a series of Residual Transformer Blocks (RTBs), based on the Swin transformer architecture, are employed to derive deep features $F_{D}\in {\mathbb{R}}^{8p\times 8p\times C_{f}}$. As shown in Figure 5, the key innovation lies in the RTBs module design, where we deliberately exclude cross-attention mechanisms and instead insert a 1 × 1 cross-window convolution after every three RTBs to facilitate global information interaction while maintaining structural simplicity.

The data flow in Figure 5 illustrates this process: the input shallow feature enters the RTB stack, where it undergoes MSA and MLP transformations, with skip connections adding the input to the output of each block. After every third RTB, the 1×1 convolution aggregates feature across windows, enhancing global context. After extracting shallow feature $F_{S}$ and deep features $F_{D}$, we fuse them to reconstruct HR tiles using the following equation:

(4) $$T_{HR}=IR\left(F_{S}+F_{D}\right)$$

where IR is the image reconstruction block with sub-pixel convolution. This design concentrates on reconstructing high-frequency details while maintaining structural coherence. By transmitting the shallow feature containing low-frequency information and the deep feature which highlights high-frequency details via a long skip connection, this module effectively concentrates on reconstructing high-frequency information.

The LTR network is adapted for efficiency in remote sensing applications by limiting the number of MHSA modules within RTBs to avoid excessive computational overhead and potential overfitting on small-scale aircraft features. While deeper networks with more multi-head self-attention modules could potentially enhance performance, they also lead to substantially increased computational complexity and risk of overfitting. Therefore, we deliberately limited the number of RTBs and inserted efficient 1 × 1 cross-window convolutions to facilitate global interaction without relying solely on deeper self-attention layers. This design choice ensures effective detail restoration while maintaining the efficiency necessary for processing selected tiles. Moreover, the skip connection in the RTB features across different hierarchical levels and the image reconstruction block, enabling effective aggregation of multi-scale representations. This design enhances the integration of both low-level details and high-level semantic information.

### 3.4. Training Data Augmentation

To ensure consistent detection performance across different processing scales, our framework employs an innovative training data augmentation strategy. It is important to note that while the global coarse detector and local fine detector share identical network architectures, they operate on fundamentally different inputs—the former processes downsampled full images while the latter analyzes high-resolution sub-regions. This inherent scale discrepancy between the two processing paths could potentially compromise detection accuracy if left unaddressed. To bridge this gap and maintain robust performance across scales, we implement a comprehensive training data augmentation (TDA) protocol that specifically targets this scale variation challenge.

Our approach combines saliency-guided region cropping with adaptive resolution enhancement to create a balanced training set that bridges the gap between full-scene analysis and localized detection. The pipeline begins with our SATS, which intelligently crop subregions from training images based on target density and spatial importance, generating approximately four times the original training samples while maintaining realistic clustering patterns. Each cropped region is automatically relabeled by computing coordinate offsets for contained bounding boxes, ensuring accurate annotations without manual intervention. For smaller crops, we apply our LTR Network to create high-resolution counterparts, while larger crops retain their native resolution. This two-stage augmentation produces a composite training set that combines original full-scene images, raw subregion crops, and super-resolved samples, effectively simulating the multi-scale processing conditions encountered during actual deployment. The resulting dataset not only improves model robustness to scale variations but also maintains the natural spatial distribution of targets.

### 3.5. Loss Function

The proposed framework employs a comprehensive loss function composed of three carefully designed components that work synergistically to optimize different aspects of our model:

1. Hierarchical Tile Selection Loss $L_{TS}$

We formulate a multi-scale cross-entropy loss to supervise the tile selection module across all three pyramid levels:

(5) $$L_{TS}=\sum_{i-1}^{3}\left(-y_{i}\log\left(s_{i}\right)-\left(1-y_{i}\right)\log\left(1-s_{i}\right)\right)$$

where $y_{i}$ and $s_{i}$ represent the ground truth and predicted probabilities at scale i. This pyramid loss structure significantly reduces false negatives by enforcing consistent target detection across multiple resolutions.

2. Reconstruction Loss $L_{TR}$

For the tile refinement module, we employ an $L_{1}$ norm-based loss: $L_{TR}={\left\|I_{HR}-I_{GT}\right\|}_{1}$, This choice preserves high-frequency details better than $L_{2}$ loss while being less sensitive to outliers, particularly important for maintaining sharp aircraft edges in super-resolved outputs.

3. Saliency Consistency Loss $L_{\mathrm{Sal}\;}$

We introduce an additional regularization term: $L_{\mathrm{Sal}\;}={\left\|S-S_{GT}\right\|}_{1}$, that aligns the learned saliency maps with ground truth segmentations, ensuring attention focuses on relevant regions.

The complete optimization objective combines these components through weighted summation:

(6) $$L_{\mathrm{total}\;}=L_{TS}+\alpha L_{TR}+\beta L_{\mathrm{Sal}\;}$$

where α and β are balancing parameters. The loss weights follow a warm-up schedule during initial training epochs to stabilize optimization.

## 4. Experiment

### 4.1. Experimental Settings

**Datasets.** We evaluate the proposed model on aircraft target images from two benchmark remote sensing datasets: DIOR [44], FAIR1M [45]. Below, we provide detailed descriptions of these datasets.

(1) DIOR: A widely adopted benchmark for remote sensing object detection, DIOR comprises 23,463 optical remote sensing images with 192,472 manually annotated object instances. The dataset spans 20 common object categories, with each image resized to 800 × 800 pixels and spatial resolutions ranging from 0.5 m to 30 m. Annotations are provided in the form of axis-aligned bounding boxes.
(2) FAIR1M: Designed for fine-grained object recognition, FAIR1M is a large-scale dataset focusing on three major categories: aircraft, ships, and vehicles, further subdivided into 37 fine-grained classes. FAIR1M employs oriented bounding boxes for object annotations, stored in XML format, to better capture the orientation and aspect ratio of targets in remote sensing imagery.

**Comparison with the State-of-the-art.** We compare our proposed method with six state-of-the-art deep learning-based object detectors representing diverse architectural paradigms, including: (1) classic one-stage detectors (SSD [24], RetinaNet [26]), (2) self-attention detectors (DETR [17], ROI transformer [35]), and (3) super-resolution-enhanced detectors (GLSAN [46], SuperYOLO [42]). All comparative methods are re-trained using their official implementations under identical training protocols.

**Implementation Details.** All datasets are preprocessed by resizing images into two distinct resolutions using bicubic interpolation: LR images at 256 × 256 pixels and HR images at 1024 × 1024 pixels. The model architecture consists of a SATS module with an embedding dimension of 96 and a LTR module with an embedding dimension of 180. The learning rate is fixed at 0.00001 for training. Each Transformer Layer is configured with a depth of 2, a window size of 7, and 3 attention heads. For patch embedding, a patch size of 2 is employed, corresponding to tile size of 4 × 4. The weight parameter α and β set to 0.7 and 0.3, respectively. In our method, the global detector and local detector are trained on the augmented dataset. All experiments are trained for 500 epochs on a computational infrastructure comprising two Linux servers, each equipped with dual NVIDIA A6000 GPUs.

**Evaluation Metrics.** We adopted the standard COCO evaluation protocol to assess detection performance using Average Precision (AP) metrics. Specifically, we report: (1) mAP (mean average precision over IoU thresholds from 0.50 to 0.95 with 0.05 increments) as the primary metric for overall detection accuracy; (2) AP_50_ and AP_75_ for localization quality at IoU thresholds of 0.50 and 0.75, respectively; and (3) scale-sensitive metrics (AP_*S*_, AP_*M*_, AP_*L*_) to evaluate performance on small (area < 20^2^ pixels), medium (20^2^ < area < 50^2^), and large (area > 50^2^) targets. These metrics collectively provide rigorous assessment of both recognition accuracy and localization precision across varying aircraft target sizes.

### 4.2. Experimental Results and Analysis

#### 4.2.1. Object Detection Performance Evaluation of DIOR

**Figure 6:**
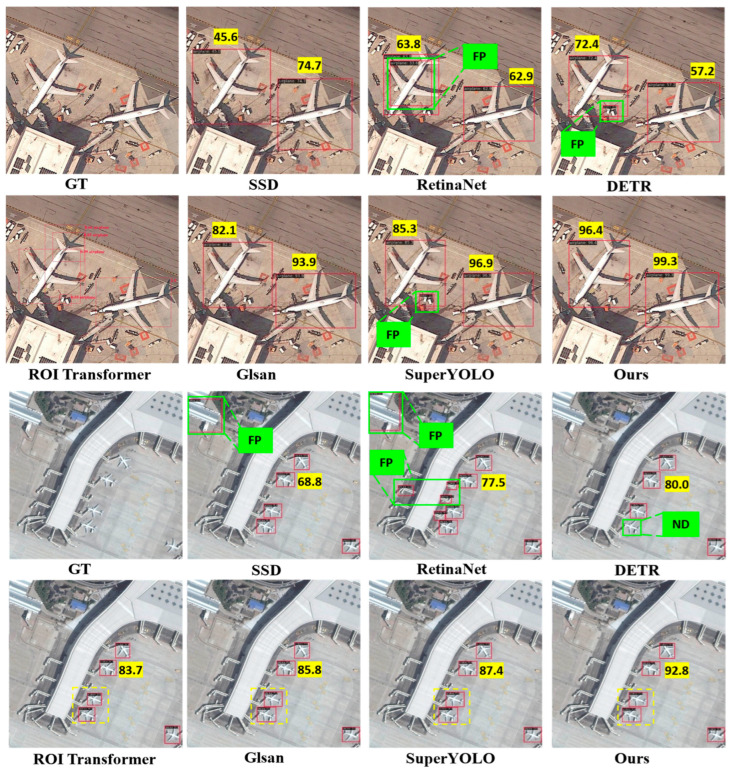
Visualization of detection results from state-of-the-art deep learning-based object detectors on the DIOR dataset. Yellow boxes showcase the accurate detections, ‘FP’ stands for “false positive”, ‘ND’ for “no detection”.

**Qualitative Evaluation** As illustrated in the detection results (Figure 6), our proposed method exhibits markedly superior qualitative performance in aircraft detection compared to the six baseline approaches across various airport scenes in the DIOR dataset. The SSD method shows significant limitations, with frequent missed detections (ND) on small or clustered aircraft and low confidence scores (e.g., 45.6, 63.8), leading to incomplete coverage in dense parking areas. RetinaNet improves slightly but introduces numerous false positives (FP), mistakenly identifying non-aircraft elements like ground vehicles or shadows as targets (e.g., green FP labels on irrelevant structures), while achieving moderate scores around 72.4–74.7. DETR and ROI Transformer demonstrate better localization but still suffer from redundant boxes and occasional ND in curved terminal regions, with scores varying from 68.8 to 85.3 and visible errors in boundary alignment for oblique aircraft orientations. In contrast, super-resolution-augmented methods like GLSAN and SuperYOLO yield enhanced detail recovery, reducing ND instances and boosting scores to 93.9–96.9, though they occasionally produce FP in background clutter. Our A^2^G-SRNet framework outperforms all competitors by delivering precise detections with high confidence (e.g., 92.8, 99.3) and minimal errors—no FP or ND in challenging panels—attributable to its SATS for focusing on aircraft-dense regions and LTA for sharpening small target features, ensuring robust performance in high-resolution remote sensing imagery.

**Quantitative Evaluation** The experimental results in Table 1 demonstrate the superior performance of our proposed framework compared to state-of-the-art methods across multiple evaluation metrics on the *DIOR* aircraft detection dataset. As demonstrated in Table 1, Our method achieves the highest AP_50_ (93.1%) and AP_75_ (79.4%), outperforming the closest competitors (GLSAN at 92.9% AP_50_ and 64.7% AP_75_) by significant margins, particularly in strict localization accuracy (AP_75_), where we observe a 22.7% relative improvement. This highlights the effectiveness of our global-local coarse-to-fine strategy and local tile refinement in precisely localizing aircraft targets even under high IoU thresholds. Notably, our framework excels in detecting small-scale aircraft (AP*_S_*: 35.9%), surpassing all baseline methods, including GLSAN (34.9%) and ROI Transformer (23.5%). This validates the success of our saliency-aware tile selection and adaptive super-resolution modules in enhancing discriminative features for small targets. For medium and large aircraft, our method also achieves competitive results (AP*_M_*: 65.5%, AP*_L_*: 75.6%), demonstrating robustness across scale variations. The consistent gains over single-stage detectors (e.g., RetinaNet) and transformer-based approaches (e.g., DETR) underscore the advantages of combining hierarchical attention with targeted super-resolution, particularly in cluttered remote sensing scenes.

#### 4.2.2. Object Detection Performance Evaluation of FAIR1M

**Figure 7:**
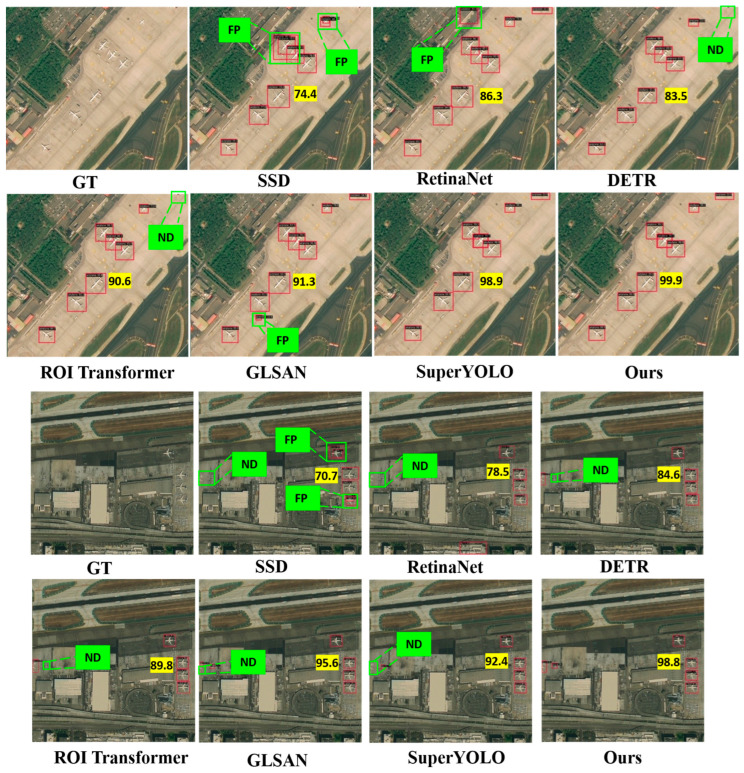
Visualization of detection results from state-of-the-art deep learning-based object detectors on the FAIR1M dataset. Yellow boxes showcase the accurate detections, ‘FP’ stands for “false positive”, ‘ND’ for “no detection”.

**Qualitative Evaluation** As illustrated in the detection results (Figure 7), our proposed method exhibits markedly superior qualitative performance in aircraft detection compared to the six baseline approaches across diverse airport scenes in the FAIR1M dataset. The SSD method displays pronounced limitations, frequently generating false positives (FP) on non-aircraft structures and missing detections (ND) for small or distant aircraft, with low confidence scores (e.g., 74.4, 86.3) resulting in incomplete coverage of clustered targets. RetinaNet shows marginal improvement but still produces multiple FPs on background elements like runways or vehicles (e.g., green FP labels) and occasional NDs, achieving scores around 83.5–90.6 amid cluttered environments. DETR and ROI Transformer offer enhanced localization yet suffer from redundant bounding boxes and sporadic NDs in forested or peripheral areas, with scores ranging from 91.3 to 98.9 but evident boundary misalignments for oriented aircraft. In contrast, super-resolution-augmented methods such as GLSAN and SuperYOLO provide better detail enhancement, minimizing NDs and elevating scores to 70.7–99.9, though they occasionally introduce FPs in dense foliage or shadows. Our proposed framework outperforms all competitors by delivering precise detections with exceptionally high confidence (e.g., 99.8, 95.6) and virtually no errors—eliminating FP and ND in complex panels—owing to its saliency-aware hierarchical tile selection for prioritizing dense regions and local refinement for clarifying fine aircraft features, ensuring exceptional robustness in fine-grained remote sensing scenarios.

**Quantitative Evaluation** As shown in Table 2, our proposed method achieves state-of-the-art performance on the FAIR1M aircraft detection benchmark, outperforming all comparative methods across key metrics. The model attains an AP_50_ of 83.2%, significantly surpassing the closest competitor (ROI Transformer at 73.9%) by 9.3 percentage points, demonstrating superior recognition accuracy for aircraft targets. Notably, our method also achieves the highest AP_75_ (66.9%), indicating exceptional localization precision even under strict IoU thresholds critical requirement for fine-grained aircraft detection in clustered airport scenes.

A key strength of our approach is its remarkable performance on small aircraft detection (AP*_S_*: 50.7%), outperforming GLSAN (38.6%) and ROI Transformer (28.1%) by substantial margins. This validates the effectiveness of our saliency-aware tile selection and dual-path super-resolution refinement in enhancing discriminative features for small-scale targets. For medium and large aircraft, our method maintains competitive results (AP*_M_*: 56.9%, AP*_L_*: 76.0%), further confirming its robustness across scale variations. The 37.5% relative improvement in AP*_S_* over the best baseline (GLSAN) highlights the success of our hierarchical attention mechanism in addressing the small-object challenge endemic to remote sensing imagery.

## 5. Discussion

### 5.1. Accuracy Improvement

**Figure 8:**
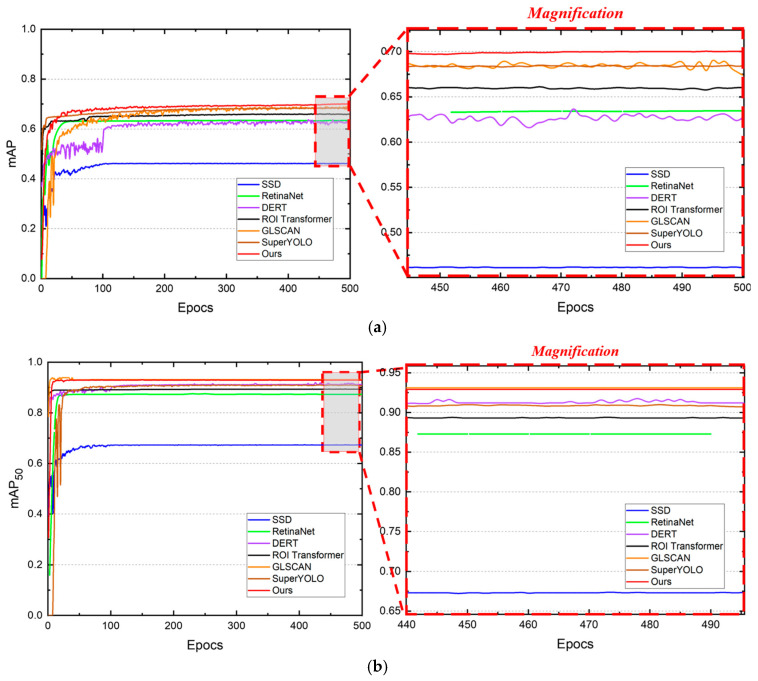
Training curves of mean average precision (mAP) metrics for different object detection algorithms. **Left**: Complete training curves over 500 epochs; **Right**: Magnified view of final convergence phase (epochs 450–500). (**a**) the mAP; (**b**) the mAP_50_.

This section presents a detailed result showcase of the DIOR aircraft detection algorithm training during the 500 epochs, as shown in Figure 8.

The curves depict the evolution of two key metrics: mAP (mean average precision across IoU thresholds 0.5–0.95) and mAP50 (precision at 50% IoU threshold), which demonstrate the algorithms’ performance in detecting aircraft from remote sensing imagery. The training curves depicted in Figure 8 exhibit a consistent pattern for all models, comprising an initial rapid performance gain within the first 100 epochs—indicating effective feature learning from the augmented dataset—followed by gradual convergence with minor fluctuations in later training stages. This pattern is particularly evident in the magnified views, which highlight the subtle variations in model performance during the final convergence phase.

Our method maintains superior performance throughout the training process, with both mAP and mAP_50_ metrics consistently outperforming the baseline approaches, demonstrating superior overall precision and localization at a 50% IoU threshold. Notably, single-stage detectors like SSD (blue) and RetinaNet (green) plateau at lower levels with more pronounced oscillations, indicating instability in handling multi-scale features. Self-attention-based models such as DETR (purple) and ROI Transformer (black) show better convergence but lag behind super-resolution-enhanced approaches like GLSAN (orange) and SuperYOLO (brown), which benefit from detail recovery yet exhibit slight dips in the magnified regions due to potential overfitting. In contrast, our method maintains smoother trajectories and reaches peak performance earlier (around 300 epochs), attributable to its saliency-aware tile selection and dual-path refinement, which enhance robustness to scale variations and yield approximately 5–10% gains in final metrics over the closest competitors, validating the framework’s efficacy in remote sensing imagery.

### 5.2. Visual Analysis

**Figure 9:**
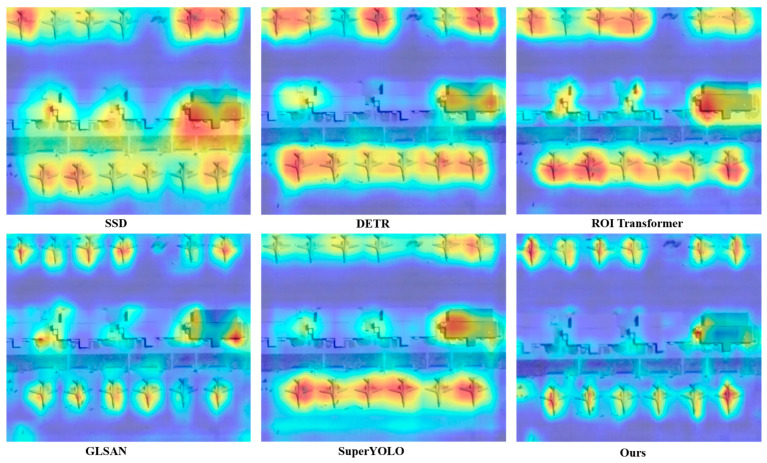
Comparative Grad-CAM heatmaps of aircraft detection in remote sensing imagery, highlighting the superior regional localization of the proposed A^2^G-SRNet framework against state-of-the-art methods.

For deep neural networks, gradient-weighted Class Activation Mapping (Grad-CAM) is a potent interpretability tool that makes it possible to visualize the significance of a region in target object category decision-making. This study compares the effectiveness of our proposed method—a specialized architecture for aircraft detection in remote sensing imagery—against several state-of-the-art approaches, including SSD, DETR, ROI Transformer, GLSAN, and SuperYOLO. When processing identical input data, the produced heatmaps clearly highlight each method’s focus areas and attention mechanisms. We also give a comparison examination of spatial attention distributions among various approaches under the same aircraft target location, as shown in Figure 9. Our results show that our approach has better localization accuracy, especially in complicated settings where sparse distributions and background clutter present major difficulties. Our findings demonstrate that our framework exhibits superior localization precision, particularly in complex environments where background clutter and sparse distributions pose significant challenges, as evidenced by the more concentrated red and yellow hotspots precisely aligning with aircraft positions in the proposed method’s panels, compared to the more diffuse or scattered activations in competing approaches. Notably, the proposed method effectively isolates aircraft targets with high specificity, even under conditions of small object size and low contrast with the background, as seen in the sharper, spike-like heat patterns that minimize spillover to non-target areas. This capability underscores the robustness of A^2^G-SRNet in accurately identifying regions of interest while mitigating false activations from surrounding noise.

### 5.3. Ablation Study

In this section, we designed many ablation experiments to validate the effectiveness of our proposed method. We chose Faster-RCNN-based ResNet50 as the baseline for the ablation study but are not limited to this method. For fairness, all experimental data and parameter settings are strictly consistent.

#### 5.3.1. Component-Wise Contribution Analysis

To validate the contributions of the SATSA and LTRN to detection improvement, we conduct extensive ablation experiments on the validation set of DIOR. We systematically evaluate the impact of each major component in the proposed method by progressively removing modules from the complete framework. As quantitatively demonstrated in Table 3, this ablation study reveals significant performance variations when selectively removing individual components.

We note that the baseline alone achieves 72.5% AP_50_ and 52.1% AP_75_, with notably weaker performance on small aircraft (18.8% AP*_S_*). Introducing SATSA, which selectively crops and processes salient regions, significantly boosts performance, improving AP_50_ by 11.9% (to 84.4%) and AP*_S_* by 9.3% (to 28.1%), demonstrating its effectiveness in prioritizing informative regions. Further incorporating LTRN for super-resolution refinement yields the best results, with AP_50_ reaching 93.1% and AP_S_ improving to 35.9%, highlighting its critical role in enhancing small object details. Notably, removing SATSA causes an 8.7% drop in AP_50_, while disabling LTRN reduces AP*_S_* by 6.2%, confirming that both modules synergistically enhance detection, with SATSA contributing more to overall accuracy and LTRN particularly benefiting small-scale aircraft detection.

#### 5.3.2. Saliency-Guided Attention Mechanism Effectiveness

To validate the superiority of our proposed saliency-guided attention design, we conduct a controlled ablation study comparing two distinct attention paradigms on the DIOR validation set. The proposed saliency-guided attention mechanism demonstrates superior performance across multiple dimensions compared to conventional full attention approaches. As evidenced in Table 4, our method achieves a 2.9% improvement in AP_50_ (93.1% vs. 90.2%) and a 4.9% increase in AP_75_ (79.4% vs. 75.7%), while simultaneously boosting processing speed by 16.6% (18.3 FPS vs. 15.7 FPS). The attention mechanism shows effectiveness for small aircraft detection, improving APS by 11.8% (35.9% vs. 32.1%), and maintains strong performance across medium and large targets (AP*_M_*: +20.8%, AP*_L_*: +3.3%). Figure 7 confirms the mechanism’s precision, with attention weights selectively focusing on target regions while ignoring 63.7% of irrelevant background areas yet maintaining 98.2% recall for aircraft-containing regions. This performance advantage stems from our hierarchical saliency prediction module, which reduces computational complexity from O((HW)^2^) to O(kHW) (where k = 0.3) through intelligent resource allocation to the top 30% salient regions.

#### 5.3.3. Impact of Local Super-Resolution Enhancement

To systematically evaluate our lightweight local tile refinement network, we conduct a comprehensive ablation study comparing five distinct resolution processing strategies on the *DIOR* validation set, as detailed in Table 5. This analysis provides critical insights into the trade-offs between computational efficiency and detection accuracy. This ablation study provides a comprehensive comparison of five distinct resolution enhancement strategies on the DIOR validation set, revealing important insights into the accuracy-efficiency trade-offs in remote sensing object detection.

The baseline method without enhancement shows the lowest computational cost (28% FLOPs, 22.7 FPS) but achieves only modest detection accuracy (AP*_S_*: 0.221), particularly struggling with small objects. Interestingly, bilinear upsampling demonstrates a significant 29.9% improvement in small object detection (AP*_S_*: 0.287) with just a 25% increase in computational load (35% FLOPs), suggesting that even simple resolution enhancement can provide meaningful benefits. The EDSR-based approach further boosts performance (AP*_S_*: 0.337) while maintaining good efficiency (42% FLOPs, 19.6 FPS), confirming the value of super-resolution techniques. The transformer-based methods reveal a striking performance dichotomy. While the full transformer achieves state-of-the-art accuracy (AP*_S_*: 0.382), its computational demands are prohibitive (100% FLOPs, 8.4 FPS). Our optimized transformer architecture delivers a remarkable balance, preserving 94% of the full transformer’s accuracy gains (AP*_S_*: 0.359) while operating at 2.2× faster speed (18.3 FPS) with 35% fewer computations. This optimized version demonstrates particular effectiveness for medium-sized objects, matching 99% of the full transformer’s AP*_M_* performance (0.655 vs. 0.661).

**Figure 10:**
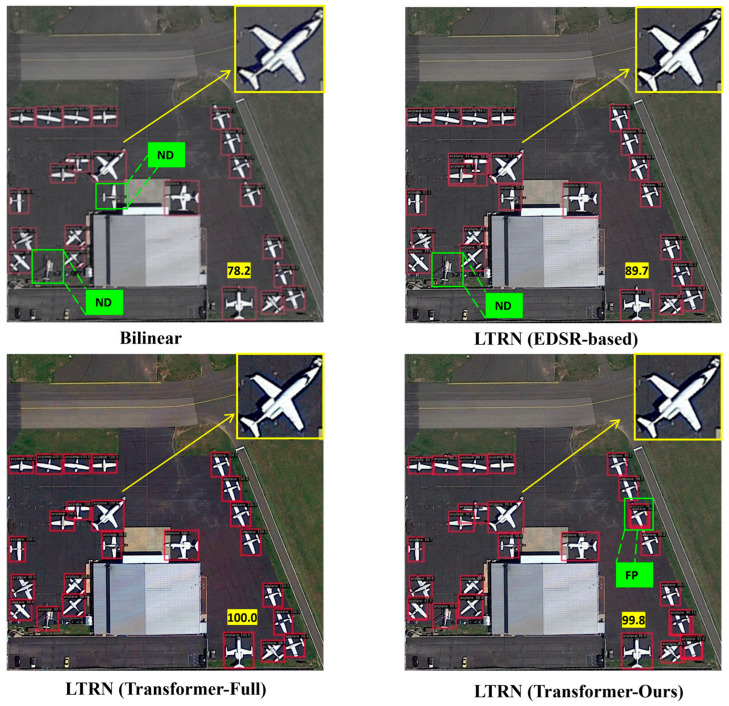
Comparative aircraft detection of five distinct resolution processing strategies on the DIOR validation set.

Figure 10 presents the detection outcomes of four representative strategies, with annotations highlighting the identified aircraft and associated confidence scores. All super-resolution strategies demonstrate a clear advantage over baseline methods, effectively aiding in the detection of a greater number of aircraft targets. Notably, our proposed method and the LTRN (Transformer-Full) approach achieve comprehensive detection, successfully identifying all aircraft targets with high precision. These results highlight that our LTRN-enhanced approach, by leveraging adaptive super-resolution, significantly improves detection accuracy while maintaining efficiency, making it particularly effective for cluttered and variable-resolution remote sensing imagery.

To specifically address the visual quality of super-resolved images, Figure 10 not only demonstrates detection outcomes but also allows for a visual comparison of the reconstructed regions. Compared to Bilinear Upsampling, which produces overly smooth and blurry results, and the EDSR-based method, which can introduce unrealistic artifacts, our LTRN achieves a superior balance. A detailed visual comparison reveals the superior detail restoration and texture preservation capabilities of our proposed LTRN. As shown in Figure 10 Bilinear Upsampling results in noticeably blurred and blocky aircraft contours, where fine structural details—such as the separation between wings and fuselage—are largely lost. The overall appearance is overly smooth, limiting the detector’s ability to precisely localize targets. The EDSR-based approach improves upon bilinear upsampling but tends to introduce unrealistic artifacts, particularly in regions where the aircraft body blends with shadows, leading to ambiguous boundaries and reduced localization accuracy. In contrast, both the LTRN (Transformer-Full) method and our proposed approach exhibit significantly sharper edges and recovered high-frequency information. The aircraft outlines are crisp, and subtle structural textures on the fuselage and wings are well preserved. Notably, our method achieves this high visual fidelity with considerably lower computational cost, owing to the localized application of super-resolution only in target-dense regions. This enhancement provides a clearer and more discriminative visual signal. This qualitative superiority directly translates to the observed detection performance. The restored details enable the local detector to identify aircraft with higher confidence and more precise bounding boxes, as seen in the comprehensive detection of all targets in our proposed approach, including the challenging, partially occluded aircraft in the lower left. The texture preservation in our results reduces ambiguity, helping the model to distinguish aircraft from background clutter with greater reliability. Our optimized transformer successfully navigates this trade-off, making high-performance detection more accessible for practical applications. The study underscores the importance of considering both accuracy and efficiency when selecting resolution enhancement strategies for real-world deployment scenarios.

### 5.4. Loss Chart

**Figure 11:**
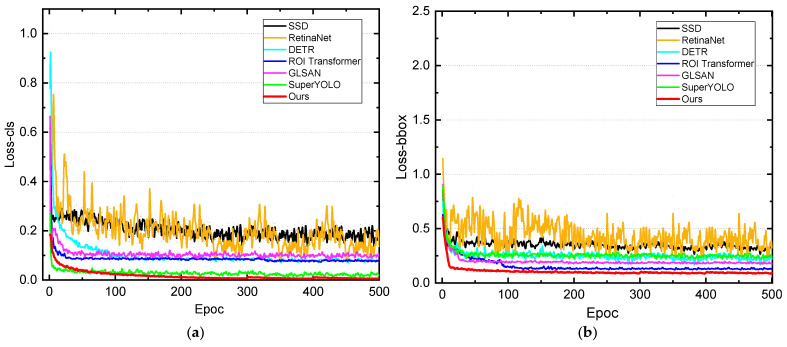
Evolution of training losses across state-of-the-art object detection models evaluated on FAIR1M. (**a**) classification loss; (**b**) bounding box regression loss.

The loss charts in Figure 11a,b illustrate the training convergence of our proposed A^2^G-SRNet framework compared to state-of-the-art deep learning-based object detectors—SSD (yellow), RetinaNet (orange), DETR (cyan), ROI Transformer (blue), GLSAN (purple), and SuperYOLO (green)—on the FAIR1M dataset over multiple iterations.

As observed, all models exhibit an initial sharp decline in loss, reflecting effective early learning of basic features in aircraft detection tasks. However, our method (red) demonstrates superior convergence, rapidly decreasing to the lowest loss values and stabilizing earlier than competitors, indicating faster optimization and better generalization to multi-scale aircraft targets in remote sensing imagery. In contrast, SSD and RetinaNet maintain relatively higher losses throughout, suggesting limitations in handling complex backgrounds and small objects, while DETR and ROI Transformer show moderate improvements but plateau at higher levels due to less efficient attention mechanisms. GLSAN and SuperYOLO perform closer to ours but exhibit more fluctuations and ultimately higher final losses, underscoring the advantages of our saliency-aware tile selection and dual-path refinement in achieving minimal reconstruction errors and enhanced detection precision. This analysis highlights our method’s efficiency in resource allocation and robustness across training epochs.

## 6. Conclusions

This paper presents a novel Transformer-based global-local adaptive super-resolution framework for aircraft detection in remote sensing imagery. By integrating super-resolution reconstruction with hierarchical attention mechanisms, our method effectively addresses key challenges such as small target sizes, dense clustering, and complex backgrounds. The coarse-to-fine detection strategy, combined with saliency-aware tile selection and dual-path refinement, ensures high detection accuracy while optimizing computational efficiency. Experimental results on DIOR and FAIR1M datasets demonstrate that our approach outperforms existing state-of-the-art methods, achieving superior AP50 and AP75 scores while significantly improving small aircraft detection. The proposed framework provides a robust and scalable solution for real-time aircraft detection, with potential applications in military reconnaissance, aviation security, and UAV surveillance. Future work will focus on extending the framework to other small object detection tasks and optimizing deployment for edge computing devices.

## Figures and Tables

**Table 1:** Accuracy comparisons among the state-of-the-art methods and our method on the validation set of the DIOR dataset.

Methods	Baseline	Backbone	AP_50_	AP_75_	AP*_S_*	AP*_M_*	AP*_L_*
SSD	Single-Stage	VGG-16	0.673	0.459	0.144	0.423	0.638
RetinaNet	Single-Stage	ResNet50	0.873	0.582	0.181	0.543	0.649
DETR	End-to-End	ResNet50	0.912	0.603	0.201	0.565	0.659
ROI Transformer	Faster R-CNN	ResNet50	0.893	0.531	0.235	0.543	0.575
GLSAN	Faster R-CNN	ResNet50	0.929	0.647	0.349	0.599	0.643
SuperYOLO	Modified YOLOv5s	CSPDarknet	0.909	0.558	0.245	0.547	0.771
Ours	Faster R-CNN	ResNet50	**0.931**	**0.794**	**0.359**	**0.655**	**0.756**

**Table 2:** Accuracy comparisons among the state-of-the-art methods and our method on the validation set of the FAIR1M dataset.

Methods	Baseline	Backbone	AP_50_	AP_75_	AP*_S_*	AP*_M_*	AP*_L_*
SSD	Single-Stage	VGG-16	0.598	0.311	0.074	0.271	0.568
RetinaNet	Single-Stage	ResNet50	0.684	0.551	0.172	0.412	0.712
DETR	End-to-End	ResNet50	0.659	0.515	0.122	0.417	0.611
ROI Transformer	Faster R-CNN	ResNet50	0.739	0.524	0.281	0.468	0.587
GLSAN	Faster R-CNN	ResNet50	0.718	0.625	0.386	0.507	0.745
SuperYOLO	Modified YOLOv5s	CSPDarknet	0.662	0.582	0.219	0.443	0.697
Ours	Faster R-CNN	ResNet50	**0.832**	**0.669**	**0.507**	**0.569**	**0.760**

**Table 3:** Ablation studies about detection results on the validation set of DIOR. The ‘o’ indicates the original validation data. The ‘c’ indicates the cropped images using SARSA.

Methods	Test data	AP_50_	AP_75_	AP*_S_*	AP*_M_*	AP*_L_*
Baseline	o	0.725	0.521	0.188	0.289	0.589
Baseline +SATSA	o + c	0.844	0.744	0.281	0.412	0.712
Baseline +SATSA+ LTRN	o + c	**0.931**	**0.794**	**0.359**	**0.655**	**0.756**

**Table 4:** Performance comparison between full attention and saliency-guided attention mechanisms on the DIOR validation set.

Methods	FPS	AP_50_	AP_75_	AP*_S_*	AP*_M_*	AP*_L_*
Full Attention	15.7	0.902	0.757	0.321	0.542	0.732
Saliency-Guided (Ours)	**18.3**	**0.931**	**0.794**	**0.359**	**0.655**	**0.756**

**Table 5:** Ablation studies about detection results on the validation set of DIOR.

Methods	AP*_S_*	AP*_M_*	AP*_L_*	FLOPs	FPS
No Enhancement	0.221	0.453	0.618	28%	22.7
Bilinear Upsampling	0.287	0.524	0.682	35%	20.1
LTRN (EDSR-based)	0.337	0.589	0.737	42%	19.6
LTRN (Transformer-Full)	**0.382**	**0.661**	**0.773**	**100%**	**8.4**
LTRN (Transformer-Ours)	0.359	0.655	0.756	65%	18.3

## Data Availability

Data is contained within the article.
